# Supplementary material for: Polyurethane Foam Incorporated with Nanosized Copper-Based Metal-Organic Framework: Its Antibacterial Properties and Biocompatibility
Source: Int J Mol Sci. 2021 Dec 19;22(24):13622. doi: 10.3390/ijms222413622 (PMC8705956; doi:10.3390/ijms222413622)
Supplement: Supplementary file 1 [file ijms-22-13622-s001.zip › ijms-1485812-supplementary.pdf]

Supplementary Material

Polyurethane Foam Incorporated with Nano-Sized Copper-Based Metal-Organic Framework: Its Antibacterial Properties and Biocompatibility

Do Nam Lee [1,*], Kihak Gwon [1,2] Yunhee Nam [3], Su Jung Lee [1], Ngoc Minh Tran [3] and Hyojong Yoo [3,*]

[1] Ingenium College of Liberal Arts (Chemistry), Kwangwoon University, Seoul 01897, Korea; khgwon@kw.ac.kr (K.G.); sue2009@live.co.kr (S.J.L.)
[2] Department of Physiology and Biomedical Engineering, Mayo Clinic, Rochester, MN 55902, USA
[3] Department of Materials Science and Chemical Engineering, Hanyang University, Ansan 15588, Korea; n2021144530@hanyang.ac.kr (Y.N.); tranminhngoc.hueuni@gmail.com (N.M.T.)

* Correspondence: donamlee2@kw.ac.kr (D.N.L.); hjhaha73@hanyang.ac.kr (H.Y.); Tel.: +82-2-940-5658 (D.N.L.); Tel.: +82-31-400-5224 (H.Y.)

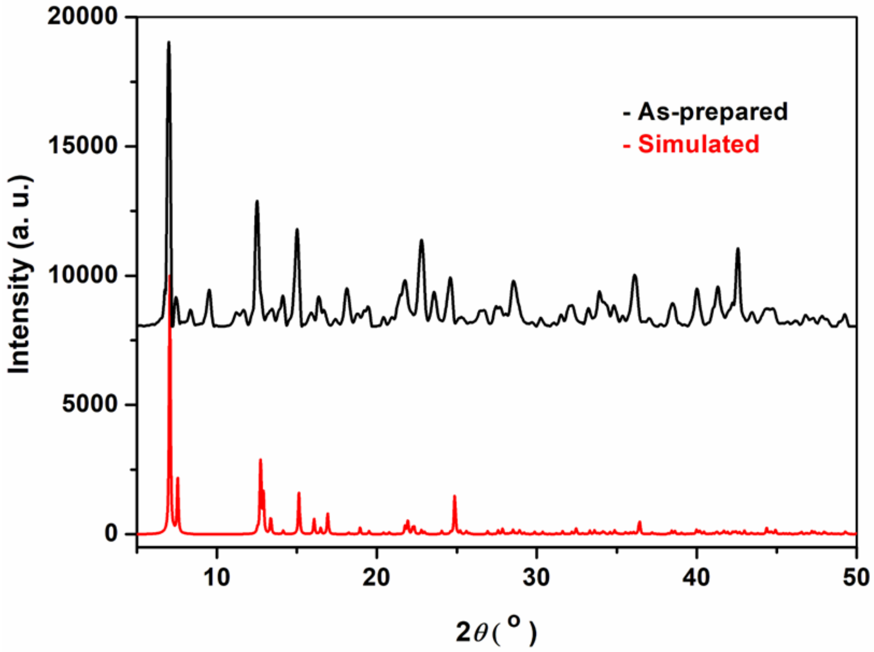

Figure S1. PXRD of simulated Cu-BTC (red) and as-prepared Cu-BTC (blue).

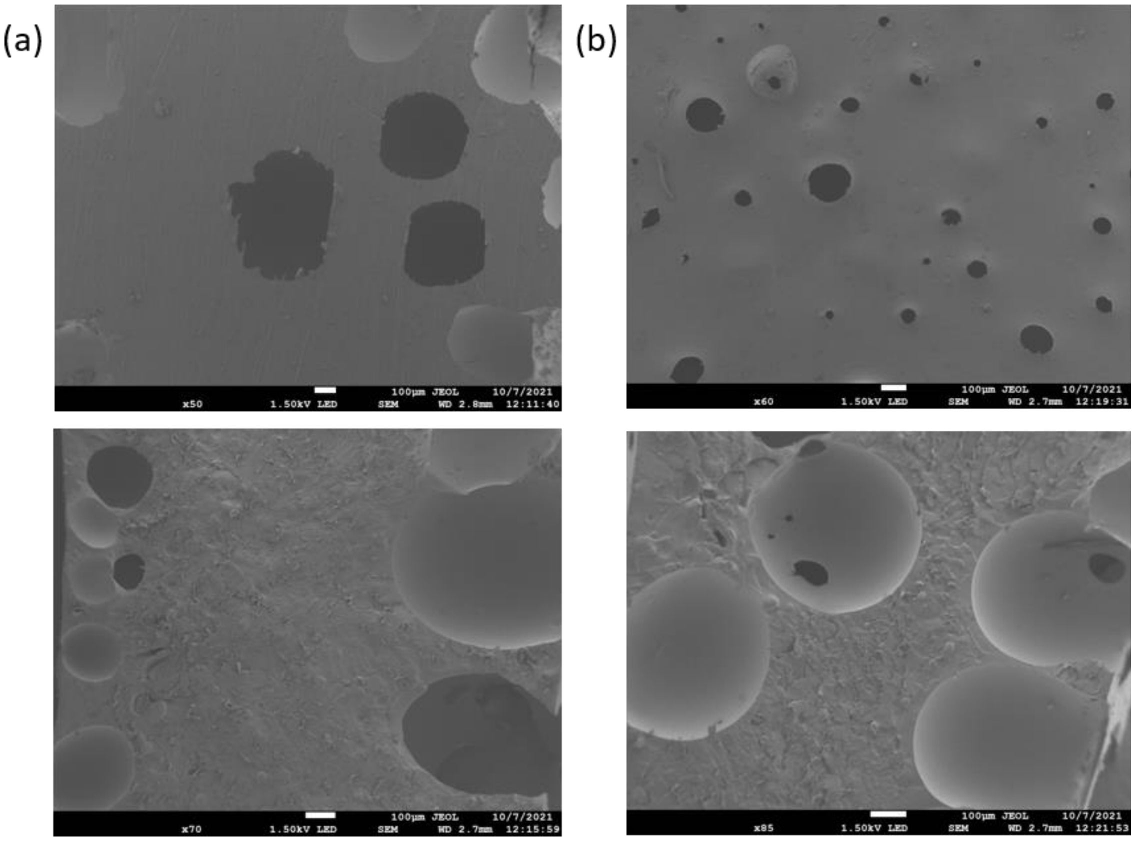

Figure S2. Scanning electron microscopy image of (a) PUF (top: upper side, under: front side) and (b) PUF@Cu-BTC (top: upper side, under: front side). Scale bar: 100 μm.
